# Protein Phosphatases in G1 Regulation

**DOI:** 10.3390/ijms21020395

**Published:** 2020-01-08

**Authors:** Ruth Martín, Vilte Stonyte, Sandra Lopez-Aviles

**Affiliations:** Norwegian Centre for Molecular Medicine (NCMM), University of Oslo. Gaustalleen 21, 0349 Oslo, Norway; r.m.martin@ncmm.uio.no (R.M.); vilte.stonyte@ncmm.uio.no (V.S.)

**Keywords:** CDK-counteracting phosphatases, Greatwall-ENSA, G1 control, Start

## Abstract

Eukaryotic cells make the decision to proliferate, to differentiate or to cease dividing during G1, before passage through the restriction point or Start. Keeping cyclin-dependent kinase (CDK) activity low during this period restricts commitment to a new cell cycle and is essential to provide the adequate timeframe for the sensing of environmental signals. Here, we review the role of protein phosphatases in the modulation of CDK activity and as the counteracting force for CDK-dependent substrate phosphorylation, in budding and fission yeast. Moreover, we discuss recent findings that place protein phosphatases in the interface between nutritional signalling pathways and the cell cycle machinery.

## 1. Introduction

Acquisition of different thresholds of cyclin-dependent kinase (CDK) activity promotes progression through the major events of the cell cycle [1,2,3]. However, in the cell, protein kinases are counteracted by protein phosphatases and it is the balance between these two activities that dictates the behaviour of the system [4,5,6]. Although this simple concept was rather intuitive, the study of protein phosphatases has been neglected for many years, on the grounds that their activity was poorly specific, given the reduced number of phosphatases compared to protein kinases and phosphorylated substrates. However, protein phosphatases belonging to the PPP family (including PP1, PP2A, PP2B, PP4, PP5, PP6 and PP7) overcome this limitation by associating to different regulatory subunits that provide localization and substrate specificity. Cdc14, PP1 and PP2A enzymes are perhaps the most widely studied phosphatases with regard to cell cycle control and we will focus this review on their roles and regulation during G1 phase. With the exception of Cdc14, which is monomeric, the other phosphatases require the interaction with specific partners that regulate their function. The catalytic subunit of PP1 (Glc7 in *Saccharomyces cerevisiae,* and Dis2 and Sds21 in *Schizosaccharomyces pombe*) associates with a variety of proteins containing the conserved motif RVxF, and this can regulate the access to substrates, as well as to specific cellular locations [7,8]. In the case of PP2A, the holoenzyme contains a catalytic subunit (Pph21 and Pph22 in *S. cerevisiae,* and Ppa1 and Ppa2 in *S. pombe*), a scaffolding subunit (Tpd3 in *S. cerevisiae* and Paa1 in *S. pombe*) and a regulatory subunit. This last component can belong to two different classes: B55 (Cdc55 in *S. cerevisiae* and Pab1 in *S. pombe*) and B56 (Rts1 in *S. cerevisiae* and Par1 and Par2 in *S. pombe*). These regulatory subunits facilitate the interaction of the complex with specific substrates and, in some instances (as we will see for PP2A-B55 enzymes), are instrumental for the regulation of the activity of the complex by signalling pathways. In the next sections we will review the events that these phosphatases control, and how they link extracellular signalling to cell cycle progression.

## 2. Phosphatases in the Regulation of CDK Activity during G1

**Figure 1:**
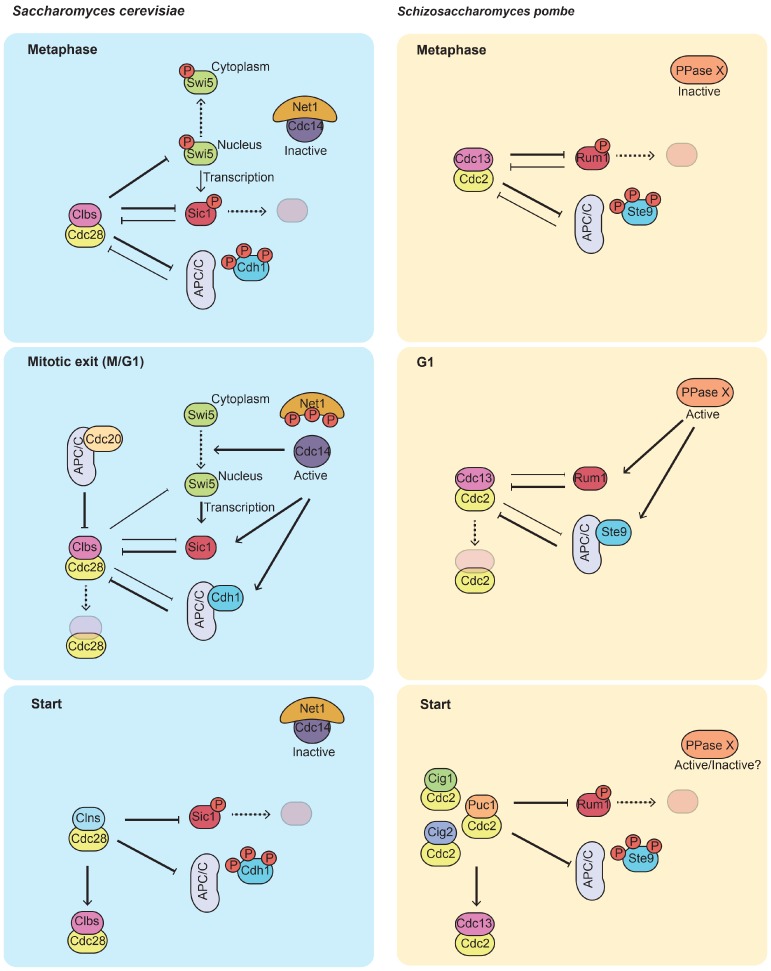
Phosphatases in the regulation of cyclin-dependent kinase (CDK) activity during G1. Left panel (budding yeast): During metaphase, high CDK activity destabilizes the CKI Sic1, prevents its transcription by Swi5 and precludes activation of the anaphase promoting complex/cyclosome (APC/C) by Cdh1. At mitotic exit, release of Cdc14 from the nucleolus, together with a decline in CDK activity leads to the accumulation of Sic1 and the activation of the APC/C^Cdh1^. Sic1 and Cdh1 sustain a state of low CDK activity during pre-Start G1 (even though Cdc14 has returned to the nucleolus). At the Start transition, the rising activity of G1 CDK complexes (which are insensitive to Sic1 and APC/C^Cdh1^) results in the degradation and inactivation of Sic1 and APC/C^Cdh1^, respectively. Right panel (fission yeast): During mitosis, high CDK activity leads to the degradation of Rum1 and prevents the binding of Ste9 to the core APC. During conditions that require a prolongation of G1 (e.g., during growth on poor nitrogen sources), Rum1 and Ste9 become dephosphorylated by an unknown phosphatase and active. This activation is reversed by G1/S-CDK complexes at the Start transition.

If high CDK (and other mitotic kinases) activity during mitosis promotes all the events required for successful chromosome segregation and cell division, during G1 CDK activity drops to its minimum. This period is essential for the cell to determine whether the external conditions are suitable for a new cycle of division, and to assess the integrity of its DNA before undergoing a new round of DNA replication. It also provides an appropriate timeframe for sexual differentiation (reviewed in [9]). CDK stoichiometric inhibitors (CKIs) and M/G1-specific co-activators of the anaphase promoting complex/cyclosome (APC/C) are integral components of the regulatory network that sustains this state of low CDK activity. In this section we will describe the events leading to their periodic activation from late mitosis until Start (summarized in Figure 1). In the budding yeast *Saccharomyces cerevisiae*, the CKI Sic1 inhibits CDK complexes containing B-type cyclins (Clb1-6 in complex with Cdc28) [10,11]. By doing so, it represses the G1/S transition, preventing initiation of replication. Sic1 is targeted for degradation through phosphorylation-dependent ubiquitination by the SCF (Skp-Cullin-F-box-containing complex) [12,13,14]. Since CDK complexes containing G1 cyclins (Cln1,2,3-Cdc28) are immune to Sic1 inhibition, those are the ones to initiate Sic1 multi-site phosphorylation and degradation during the passage through Start. Indeed, this is the most essential function of these complexes, as deletion of Sic1 is sufficient to rescue the viability of a *cln1 cln2 cln3* mutant [12,15,16]. Whilst the cell cycle proceeds and B-type cyclins accumulate, Clb-Cdc28 complexes maintain Sic1 phosphorylation and inhibition [9,17]. It is only at the end of mitosis, during the M/G1 transition that Sic1 is dephosphorylated by Cdc14 and can promote the irreversible inactivation of CDK and the establishment of a stable G1 phase [18,19,20]. In addition, Cdc14 contributes to Sic1 accumulation by facilitating its transcriptional activation; Swi5, the transcription factor responsible for Sic1 expression [21], is exported from the nucleus upon CDK phosphorylation [22] and it only becomes functional once Cdc14 is activated by the Mitotic Exit Network (MEN) pathway [18,23,24]. While this regulation of Sic1 occurs during unperturbed mitotic cycles, stress responses also impinge in its function. In particular, the stress response mitogen-activated protein kinase (MAPK) Hog1 can phosphorylate Sic1 at Thr173, a residue different to those targeted by CDK complexes, which results in its stabilization, thus facilitating the arrest in G1 when cells face osmotic stress conditions [25]. Mpk1, another MAPK, is also required for Thr173 phosphorylation and stabilization of Sic1, in this case upon inactivation of TOR complex 1 (TORC1). Notably, concomitant downregulation of PP2A-B55^Cdc55^ by the Rim15-Igo1/2 pathway (the budding yeast Greatwall-Endosulphin Alpha (ENSA) pathway) is critical for the phosphorylation and protection from degradation of Sic1 [26]. This aspect of G1 control by protein phosphatases will be further explored in the next sections.

Besides Sic1-mediated inhibition, persistent degradation of B-type cyclins also contributes to the repression of CDK activity during G1 [27]. During mitotic exit, the APC/C activator Hct1/Cdh1, takes over the control of Clb2 ubiquitination and sustains it until Start [28,29]. Like Sic1, Cdh1 is phosphorylated by CDK complexes, and this phosphorylation hinders its binding to the core APC/C subunits [30,31]. Hence, the inhibition of Cdh1 is only relieved when Cdc14 is released from the nucleolus by the MEN during anaphase [18,31]. Importantly, the APC/C-Cdh1 also targets the Polo kinase Cdc5 for degradation, which is essential for the nucleolar release of Cdc14 [32]. Therefore, once dephosphorylated, Cdh1 triggers the re-sequestration of Cdc14 and prevents its further activation. It is also worth noting that, although Sic1 and Cdh1 have important functions during G1, Cdc14 is already inactive during this phase of the cell cycle. 

In the fission yeast *Schizosaccharomyces pombe*, the counterparts of Sic1 and Cdh1, Rum1 and Ste9 are also instrumental for the inactivation of CDK (Cdc2) activity and establishment of a pre-Start G1 phase. This function, however, can only be appreciated when cells are exposed to poor nutritional conditions, in particular to the absence of a source of nitrogen. The reason for this stems from the fact that *S. pombe* cells do most of their growth during G2 phase of the cell cycle. So much so that upon mitosis and cytokinesis they have already attained the minimal size required to commit to a new round of division. Still, if cell growth during G2 phase is limited (when nitrogen is not available or in mutants that enter mitosis prematurely, e.g., *wee1* mutants) G1 phase needs to be extended. This is brought about through the engagement of an otherwise cryptic G1 cell size checkpoint that is governed by the CKI Rum1 [33]. Thus, in the presence of a poor nitrogen source or in the extreme case of nitrogen starvation, Rum1 accumulation halts the cell cycle in G1. From this point the cell can decide to enter a quiescence state or, if a mating partner is available, undergo sexual differentiation and conjugation. As it happens for budding yeast, targeted degradation of B-type cyclins by APC/C-Ste9 also contributes to CDK downregulation during G1. Furthermore, like Sic1 and Cdh1, both Rum1 and Ste9 are negatively regulated by CDK phosphorylation, leading to degradation of the former and inhibition of the binding to the APC/C of the latter [34,35,36]. Importantly, not all CDK complexes are equally sensitive to Rum1 and Ste9 activities. Rum1 inhibits CDK complexes containing the B-type cyclins Cig2 and Cdc13, while Cig1-Cdc2 or Puc1-Cdc2 are largely unaffected by its presence. Similarly, the APC/C-Ste9 ubiquitinates Cdc13 and Cig1, but cannot target Cig2 or Puc1. In consequence, the balance between these negative and positive regulators of CDK activity dictates passage through Start. Whether a phosphatase activity also participates in these events (counteracting CDK phosphorylation of Rum1 and Ste9) is still an outstanding question. The fission yeast Cdc14 homolog, Clp1/Flp1, was shown early on not to affect the accumulation and activation of Rum1 and Ste9 [37]. However, this is perhaps not surprising in light of the observations that in *S. pombe*, a phosphatase relay encompassing the activities of PP1, PP2A-B55^Pab1^ and PP2A-B56^Par1^ controls mitotic exit [38]. In fact, results from our laboratory indicate that members of the PP2A family are the most likely phosphatases opposing CDK phosphorylation on Rum1 and Ste9. 

What are the consequences of not being able to downregulate CDK activity in G1? Despite being key regulators of CDK activity, neither Sic1, nor Cdh1 are essential proteins. Nevertheless, the double mutant *sic1 cdh1* arrests in late anaphase, indicating that they act redundantly in bringing about the inactivation of CDK complexes at the end of mitosis [28]. It is worth noting that, although the individual *sic1* and *cdh1* mutants are still able to divide, they fail to restrain the build-up of CDK activity during G1 and this has deleterious consequences during S phase. Specifically, precocious activation of Clb5/6-Cdc28 complexes in these mutants causes defects in the licensing of replication origins and genomic instability [39,40]. In the case of *S. pombe*, deletion of either Rum1 or Ste9 prevents the arrest the cell cycle in G1 in response to nitrogen starvation and, in consequence, *rum1* and *ste9* mutants are sterile [33,41,42,43,44]. Moreover, a recent study has shown that, in their absence, activation of the transcriptional complex MBF (*MluI* cell cycle box-binding factor) is impaired [45], resulting in replication stress and DNA damage. Similar phenotypes would be expected in mutants of the phosphatases that counteract CDK-phosphorylation in Sic1/Rum1 and Cdh1/Ste9. In budding yeast, mutation of Cdc14 arrests cells prior mitotic exit, hindering the observation of defects in the subsequent G1 and S phases. Since, Rum1 or Ste9 are not required to exit mitosis, it might be easier to appreciate the consequences of their persistent phosphorylation in phosphatase mutant backgrounds, in fission yeast. 

While budding yeast Cdc14 promotes the degradation of B-type cyclins, PP2A-B55^Cdc55^ has been shown to promote the stabilization of the G1 cyclin Cln2 [46]. G1 cyclins (Cln1-3) are degraded at the end of G1 phase through their phosphorylation-dependent ubiquitination by the SCF [47,48,49,50,51]. This phosphorylation is carried out by Cdc28, either in complex with B-type cyclins [52] or with G1 cyclins [50]. The targeted degradation of G1 cyclins together with the transient nature of their transcriptional induction, which is repressed by G2 cyclins (Clb1-4) [53], results in their presence in the cell being confined to G1 and early S phase [54,55]. However, McCourt et al. showed that in the absence of PP2A-B55^Cdc55^ Cln2 disappearance is accelerated. The authors did not investigate the behaviour of other G1 cyclins (Cln1 and Cln3) and neither did they explore the consequences of the aberrant degradation of Cln2 in detail. Nevertheless, it would be expected that if G1 cyclins are not allowed to accumulate in the cell, transition through Start, inhibition of Sic1 and Cdh1, and activation of B-type cyclins will be compromised. 

## 3. Phosphatases Counteracting CDK-Phosphorylation in G1

In the previous section we analysed how loss of phosphatase activity impinges in the regulation of CDK complexes. Yet, protein phosphatases also affect the pattern of CDK-substrate phosphorylation by directly opposing the action of CDK complexes. The two functions are somehow difficult to separate since, as we explained before, CDK regulators are also CDK substrates. However, in this section we will focus on the role of protein phosphatases as the workhorse that cancels out CDK phosphorylation in the G1 cell. Given the short time that fission yeast cells spend in this phase of the cell cycle under normal conditions, the study of the phosphatases that counteract CDK activity during G1 is limited. In contrast, budding yeast has been a more useful model to investigate the identity of the CDK-opposing phosphatases. During mitotic exit, the balance of kinase and phosphatase (Cdc14) activities affects the order of CDK substrate dephosphorylation [4]. Nonetheless, during interphase Cdc14 is sequestered in the nucleolus and cannot access its substrates. Therefore, a different protein phosphatase has to counterbalance the increase in CDK activity (mediated by the transcriptional activation of cyclins) in order to prevent the premature phosphorylation of CDK substrates. Using SILAC-based quantitative proteomics during a synchronized cell cycle, Godfrey et al. showed that PP2A-B55^Cdc55^ opposes CDK phosphorylation during G1, S and G2 phases [6]. In the absence of PP2A-B55^Cdc55^, cells depict untimely phosphorylation of known late CDK substrates such as Sli15 or Ndd1. This study also revealed that PP2A-B55^Cdc55^ dephosphorylates phospho-threonines and phospho-serines followed by a proline (which constitutes the minimal CDK consensus), and that it portrays a preference for threonines *vs* serines. In addition, the presence of basic amino acids downstream of the phospho-site (as in the full CDK-consensus S/TPxK/R) favours the recognition of substrates by PP2A-B55^Cdc55^. These results were also confirmed in mammalian cells [56], indicating that the determinants for substrate recognition by PP2A-B55 enzymes have been preserved through evolution. The differential specificity for the phospho-acceptor residue results in the late phosphorylation of threonine-containing CDK substrates. Conversely, serine-containing substrates are less affected by the presence of PP2A-B55^Cdc55^ and hence are phosphorylated early during a normal cell cycle. Interestingly, the mitotic phosphatase Cdc14, which becomes activated upon inhibition of PP2A-B55^Cdc55^ by separase [57], has preference for phospho-serines instead. Hence, depending on the phospho-acceptor sites, the sensitivity to phosphatase activity of a substrate will be different during mitosis and during interphase. 

Is PP2A-B55 the only phosphatase counteracting CDK phosphorylation during G1? In higher eukaryotes PP1 and PP2A cooperate in the dephosphorylation of pRB in order to block passage through the restriction point, which indicates that PP1 can also oppose CDK-dependent phosphorylation [58,59]. The budding yeast PP1 phosphatase, Glc7, associated to Rif1 has been shown to counteract phosphorylation of the components of the preRC (pre-replicative complex) by DDK (Dbf4-dependent kinase), thus preventing replication origin firing [60,61,62]. Even though DDK cooperates with CDK complexes in this process, it was clear from these studies that Rif1-Glc7 role was limited to DDK-dependent phosphorylation events. Interestingly, recruitment of Glc7 was itself counteracted by CDK/DDK-mediated phosphorylation of Rif1, hence providing a mechanism of regulation of Rif1-Glc7 function. In the study by Godfrey et al. overexpression of *CDC55* delayed the phosphorylation of the Origin Recognition Complex component Orc6 by S-CDK complexes. Therefore, the two phosphatases, Glc7 and PP2A-B55^Cdc55^ would cooperate in the counteraction of DDK and CDK-dependent phosphorylation events at the replication origins.

In the case of budding yeast PP2A-B56^Rts1^_,_ a phosphoproteomic study from the Kellogg laboratory implicated this phosphatase in the control of G1-cyclins expression and Swe1 inhibition [63]. These functions are required for the control of cell size and will be explored in more detail in the next section. Nevertheless, although CDK substrates (e.g., Swi4) were among the proteins whose phosphorylation increased in cells deleted for *rts1*, the authors did not see a general pattern of phosphorylation that indicates that PP2A-B56^Rts1^ is a general CDK-counteracting phosphatase. However, one must keep in mind that proteome-wide mass spectrometry is not comprehensive, and that additional specific CDK substrates dephosphorylated by PP2A-B56^Rts1^ might exist although they were not identified in this study. 

Importantly, as new phosphoproteomic studies emerge, we are getting a clearer picture of the events controlled by individual phosphatases, as well as of the elements dictating their substrate specificity [6,56,64,65]. In the case of PP2A-B56 enzymes, they recognize a short linear motif (SLiM) encompassing the sequence LxxIxE [64,66]. When the second residue in this motif is a serine or a threonine, its phosphorylation enhances the recognition by B56. Interestingly, the third residue is often found to be a proline, which would then create the minimal consensus for CDK phosphorylation. This opens the possibility that dephosphorylation of PP2A-B56 substrates may be influenced in some instances by CDK activity.

PP1 interacting proteins (PIPs) are also characterized by a specific binding motif (RVxF) [7] that mediates the binding to the catalytic subunit. This motif is present in PP1 substrates, targeting subunits and inhibitors. Hence, as for B56-containing PP2A enzymes, it will be the presence of this specific SLiM in a substrate rather than the sequence surrounding the phospho-site that will dictate its dephosphorylation by PP1. This might be the reason why neither PP1 nor PP2A-B56 appear as general CDK-counteracting phosphatases, whereas Cdc14 and PP2A-B55 do. In the former case dephosphorylation requires the presence of an exposed SLiM, while in the latter the phospho-site itself and its neighbouring amino acids conform the motif recognised by the phosphatase (and the kinase). In consequence, PP2A-B55 might more generally dephosphorylate CDK-substrates, while PP1 and PP2A-B56 might cooperate with it in the dephosphorylation of specific SLiM-containing substrates. To confirm this idea, more studies similar to the one carried by Godfrey et al. using synchronized cultures of phosphatase mutants, together with the identification of interacting proteins, will be required. 

## 4. Nutrient Sensing Pathways in the Regulation of Phosphatase Activity in G1

Sensing of environmental conditions, in particular of nutrient availability, is intimately linked to cell cycle progression. The target of rapamycin complex 1 (TORC1) and protein kinase A (PKA) signalling pathways are key elements in the transduction of nutritional cues and the control of cell growth and division. In this section we review the interplay between these pathways and protein phosphatases, with particular emphasis on the Greatwall-ENSA pathway that specifically controls PP2A-B55 activity.

### 4.1. The Greatwall-ENSA Pathway in the Control of G1 and G0

In recent years, the discovery that the Greatwall kinase and its substrate ENSA negatively regulate the activity of PP2A-B55 has brought new interest in the study of the functions of this phosphatase [67,68,69,70]. 

**Figure 2:**
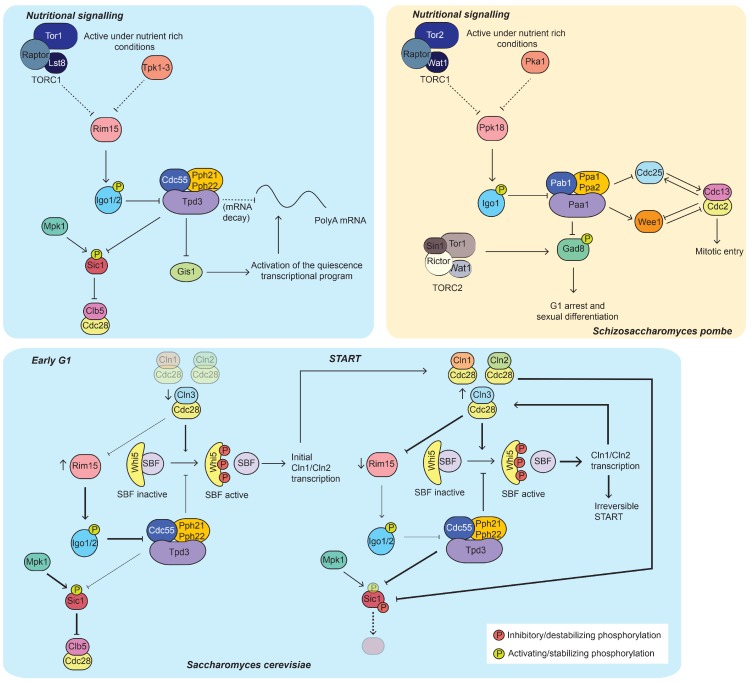
The Greatwall- Endosulphin Alpha (ENSA) pathway in the control of G1 and G0. Left panels, in blue (*Saccharomyces cerevisiae*): During growth on rich nitrogen and carbon sources, the TORC1 and PKA pathways repress Rim15, and PP2A-B55^Cdc55^ dephosphorylates and prevents Gis1-mediated transcription of quiescence genes. Upon TORC1 and PKA inhibition, Rim15 is activated and phosphorylates Igo1/2, which is a potent PP2A-B55^Cdc55^ inhibitor. As PP2A-B55^Cdc55^ is inactivated, Gis1 initiates the quiescence transcriptional program. Furthermore, inhibition of PP2A-B55^Cdc55^ favours the post-transcriptional stability of those Gis1-regulated targets. PP2A-B55^Cdc55^ also counteracts Mpk1 phosphorylation of Sic1 on Thr173. When PP2A-B55^Cdc55^ is inhibited during nutritional deprivation, Sic1 is stabilized and becomes an inhibitor of Clb5-Cdc28, facilitating the arrest in G1. This regulation also takes places in proliferating cells: during early G1, Rim15 activity is high, preventing Thr173 dephosphorylation and degradation of Sic1. At the Start transition G1 CDK complexes repress Rim15. This results in the activation of PP2A-B55^Cdc55^ and the dephosphorylation of Sic1 Thr173. Increasing CDK activity brings about multisite phosphorylation of Sic1 that triggers its degradation. In addition, the Rim15-ENSA-PP2A-B55^Cdc55^ pathway regulates the expression of SBF-dependent genes (e.g., *CLN1*, *CLN2*) through the phosphorylation of Whi5. During early G1, inhibition of PP2A-B55^Cdc55^ contributes to the initial phosphorylation of Whi5 by Cln3-Cdc28, which promotes the transcription of *CLN1* and *CLN2.* As Cln1,2-Cdc28 activity rises, Whi5 is further phosphorylated and the Start transition is made irreversible. The antagonistic relation between G1-CDK complexes and Rim15 and their cooperation in the activation of SBF contributes to the homeostatic control of Start, facilitating Whi5 phosphorylation when Cln3 levels are low during growth on poor nutritional conditions. (see main text for details) Right panel, in orange (*Schizosaccharomyces pombe*): During growth on rich nitrogen and carbon sources, TORC1 and PKA repress Rim15. This inhibition is relieved upon nutritional stress, promoting the phosphorylation of Igo1 and inactivation of PP2A-B55^Pab1^. Since PP2A-B55^Pab1^ counteracts CDK-dependent phosphorylation on Wee1 and Cdc25, its inhibition brings about the activation of CDK and mitotic entry at a smaller cell size. In turn, this leads to the engagement of the G1 cell size checkpoint and prolongation of this phase. Additionally, PP2A-B55^Pab1^ opposes TORC2 phosphorylation on its effector Gad8. Following nitrogen deprivation, PP2A-B55^Pab1^ inhibition facilitates the phosphorylation of Gad8, G1 arrest and engagement of the sexual differentiation response.

In metazoans, studies have focused in the role of this pathway in the control of mitotic entry and exit, where inhibition of PP2A-B55 by phosphorylated ENSA triggers mitotic CDK activation. However, the Greatwall homolog in budding yeast, the protein kinase Rim15, had been studied for many years as a critical regulator of the cellular responses to nutrient limitation [71]. Mutants lacking *rim15* fail to express genes required for entry into quiescence, as well as to arrest their cell cycle progression in G1 when they are grown to stationary phase. Seminal works from the de Virgilio laboratory went on to show that Rim15 is essential for life-span extension and stress resistance [72] as well as entry into quiescence upon inactivation of TOR and/or PKA [73]. These pathways and the Pho80-Pho85 CDK complex control Rim15 activity and promote its cytoplasmic sequestration through the binding to a 14-3-3 protein when growth conditions are optimal [73,74]. Conversely, Rim15 translocates to the nucleus where it becomes active following glucose limitation at the diauxic shift. This results in the engagement of a transcriptional program that leads to quiescence and that is controlled by the transcription factors Gis1, Msn2 and Msn4 [75,76,77]. Nevertheless, it took several years until the molecular mechanisms mediating the roles of Rim15 were elucidated (summarized in Figure 2, left panels). The ENSA homologs Igo1/2 were found to be essential for the initiation of the quiescence program upon TORC1 inhibition, by specifically blocking the decapping and degradation of nutrient-regulated mRNAs (e.g., *HSP26*) [78,79]. This function and the transcriptional activation of the quiescence program by Gis1 requires the Igo1/2-mediated repression of PP2A-B55^Cdc55^ [80,81]. Under poor growth conditions, activated Rim15 phosphorylates Igo1/2, which then becomes a potent inhibitor of PP2A-B55^Cdc55^ through a mechanism of unfair substrate competition [82]. Since this molecular pathway was unraveled, PP2A-B55^Cdc55^ has been revealed as instrumental for the control of G1 phase as well as for the establishment of the differentiation response [83]. PP2A-B55^Cdc55^ inhibition following TOR downregulation is required for the Mpk1-dependent phosphorylation of Sic1 at Thr173, which results in its stabilization [26]. Moreover, it facilitates the conversion of Sic1 from a substrate into an inhibitor of Clb5-CDK [84]. Notably, this function does not only occur upon silencing of the TOR pathway, but it also operates in cycling cells. Thus, in early G1 phase, high Rim15 activity precludes PP2A-B55^Cdc55^ dephosphorylation of Sic1 Thr173. At the Start transition, the rise in the activity of Cln-CDK complexes (which, as the Pho80-Pho85 complex, can phosphorylate Rim15) brings about the inhibition of Rim15 and the subsequent activation of PP2A-B55^Cdc55^. As Sic1 Thr173 becomes dephosphorylated, Cln-CDK complexes initiate the Sic1 multi-site phosphorylation that ultimately leads to its ubiquitination by the SCF and degradation. This is not the only mechanism by which the Rim15-Igo1/2 pathway regulates G1 phase. In addition, it favours the activity of the Swi4/6 cell cycle box-binding factor (SBF/E2F). Early in G1, The Rim15-Igo1/2 pathway prevents the PP2A-B55^Cdc55^-mediated dephosphorylation of the SBF inhibitor Whi5/Rb (whose phosphorylation is initiated by Cln3-Cdc28) thereby triggering the initial expression of *CLN1*/*CLN2.* As CDK activity increases (due to the expression of *CLN1* and *CLN2*), Whi5 is further phosphorylated by Cln1,2-Cdc28, which results in irreversible passage through Start [85]. Importantly, this mechanism is exploited by the cell in order to regulate cell size homeostasis during metabolic rewiring. In the presence of rich medium, *CLN3* expression is high but inhibition of Rim15 by Cln3-Cdc28, TORC1 and PKA results in high PP2A-B55^Cdc55^ activity. When conditions are poor, Rim15 repression is relieved and it blocks the activity of PP2A-B55^Cdc55^, lowering the threshold of Cln3 required for the initial phosphorylation of Whi5. 

The fission yeast homolog of the Greatwall kinase was just recently identified by the laboratory of Sergio Moreno. The Ppk18-Igo1 pathway is similarly downregulated by TORC1 and PKA signalling but, in this case, this promotes the extension of G2 phase mediated by the CDK inhibitory kinase Wee1. During growth under poor nitrogen conditions, inactivation of these pathways favours the Ppk18-dependent inhibition of PP2A-B55^Pab1^ and, consequently, Wee1 repression and activation of Cdc25. Altogether, this advances mitotic entry and the cell divides at a reduced cell size [86] (Figure 2, right panel). As we mentioned in the first section, this indirectly leads to the extension of G1, as cells that do not attain a certain size during G2 need to resume growth during this phase. Results from our laboratory revealed that, in addition to this role in the control of the G2/M transition, this pathway is also required for the engagement of the mating response. This regulation involves the activation of the TORC2 effector Gad8 upon TORC1 silencing during nitrogen starvation [87]. While TORC1 inhibits sexual differentiation, TORC2 and Gad8 are essential for the cell cycle arrest in G1 and the transcriptional activation of the differentiation program. Therefore, the Greatwall-ENSA-B55 (Ppk18-Igo1-PP2A-B55^Pab1^) pathway connects the activities of the two TOR complexes, leading to the upregulation of TORC2 signalling when TORC1 is silenced (Figure 2, right panel).

Whether Ppk18-Igo1-mediated inhibition of PP2A-B55^Pab1^ has additional consequences in other events during G1 are outstanding questions worth investigating in the future.

### 4.2. Regulation of PP2A Enzymes by the TOR-Tap42 Signalling Branch 

Early studies in budding yeast suggested that TOR signalling impinged in the activity of PP2A enzymes; similar to rapamycin treated-cells, mutants of the PP2A-related phosphatase Sit4/PP6 arrested in G1, unable to accumulate the G1 cyclins Cln1 and Cln2 [88]. Subsequently, Sit4 was shown to bind the protein Tap42, a TORC1 effector, which could also interact with other PP2A catalytic subunits (Pph21 and Pph22) independently of the regulatory subunit. This interaction was shown to be sensitive to TOR activity since it was lost upon starvation or rapamycin treatment [89]. Tap42 is indeed a direct target of TORC1 that binds Sit4 and Pph21/Pph22 when phosphorylated. Upon TORC1 inhibition, Tap42 is dephosphorylated (notably by PP2A-B55^Cdc55^) and this results in the release of these phosphatases [90]. A second level of control also regulates the binding between Tap42 and PP2A catalytic subunits. The protein Tip41 (another substrate of TORC1) blocks their interaction, but to do so it needs to be dephosphorylated [91]. Incidentally, Tip41 dephosphorylation depends on the phosphatase Sit4. 

How does binding to Tap42 affect the activity of PP2A enzymes? The favoured hypothesis for a long time was that association between Tap42 and Sit4 inactivates the phosphatase. This was supported by the observation that under nitrogen rich conditions (TORC1 active) Tap42 precludes the dephosphorylation and nuclear translocation of Gln3 by Sit4. Gln3 induces the expression of genes in the nitrogen discrimination pathway (NDP) when cells are grown in the presence of a poor nitrogen source [92]. However, when nitrogen is not limiting it is phosphorylated and anchored to the cytoplasm through the binding to Ure2 [93]. Sit4 activity was shown to be required for its release and nuclear translocation upon TORC1 inactivation. This straightforward model in which Tap42 inhibits Sit4 to prevent the activity of Gln3 was challenged later on by a study demonstrating that Tap42 is required for the rapamycin-dependent induction of the NDP [94]. Moreover, Tap42 was initially isolated as a multicopy suppressor of the *sit4-102* mutant, which would argue against its being a negative regulator of Sit4 function [89]. Indeed, it was later shown that the interaction between Tap42 and Sit4 is required for the essential functions of the latter, and that the *sit4-102* allele contains a mutation within the Tap42 binding domain that hinders their interaction [95]. Taking all these results into account, together with the fact that the dephosphorylation of Sit4 substrates upon TORC1 inhibition occurred faster than the dissociation of Tap42 and Sit4, a new model was proposed; the complex between phosphorylated Tap42 and Sit4 is active, but during growth on rich conditions it is bound to TORC1 and tethered to membrane structures where it cannot access its substrates. Upon nitrogen starvation or rapamycin treatment, the interaction between Tap42-Sit4 and TORC1 is lost and the active complex is released into the cytoplasm. The subsequent dephosphorylation of Tap42, (which occurs with slower kinetics than the release from membranes) ultimately leads to the inactivation of Sit4 [96]. However, it is worth noting that only a small portion of Sit4 (between 5–10%) binds to Tap42 [89]. Other functions of Sit4 might be controlled by the Sit4 pool that is not bound to Tap42, and that is refractory to TORC1 inhibition. 

Besides PP2A enzymes, the PP2C phosphatase Ptc1 has also been shown to be required for the normal function of the TORC1 pathway in budding yeast [97]. Cell lacking *ptc1* are hypersensitive to rapamycin and caffeine and fail to translocate Gln3 and Msn2 to the nucleus upon TORC1 inactivation, which results in the impaired expression of NDP and stress response element (STRE)-driven genes. Although the molecular consequences of losing Ptc1 function are not fully understood, these defects seem to be partly due to the increased instability of Tip41 in the *ptc1* deletion mutant. In addition, the absence of Ptc1 also affects the levels and phosphorylation status of the TORC1 effector kinase Npr1. Recent findings indicate that Npr1 participates together with the phosphatidylinositol-3-phosphate (PI-3P)-binding protein Pib2 in a negative feedback controlling TORC1 activity in response to poor nitrogen sources [98]. Whether Ptc1 function is also required for this regulation is an open question.

The Sit4 fission yeast counterpart, Ppe1, has not been involved in G1 progression, but rather in the regulation of the actin cytoskeleton and glucose utilization [99]. Nevertheless, mutation of *ppe1* was shown to be suppressed by temperature-sensitive alleles of *ssp1* and *ssp2* (CaMKK and AMPKα, respectively). Both proteins function in a linear pathway to modulate TORC1 activity during nitrogen stress, facilitating cell division at a reduced size [100]. Moreover, Ssp1 was shown to promote Ssp2 activation during growth under low glucose in a process that required the additional silencing of Ppe1 [101]. Therefore, these results indicate that, in fission yeast, Ppe1 also participates in the signalling pathways that link the energy status to cell cycle progression. 

### 4.3. Nutritional Modulation of Cell Size by PP2A-B56^Rts1^

In budding yeast, a second PP2A complex, PP2A-B56^Rts1^, has been involved in the regulation of G1 progression and growth control. *rts1*∆ cells have a delay in the accumulation of G1 cyclins and in the initiation of bud growth. More importantly, cells lacking PP2A-B56^Rts1^ activity fail to couple their growth to nutritional conditions [102]. These defects in cell cycle progression are partly the consequence of impaired transcriptional control of *CLN3*, *CLN1* and *CLN2.* Importantly, PP2A-B56^Rts1^ regulates the phosphorylation status of the transcription factors Ace2 and Swi4 (that are hyperphosphorylated in the absence of *rts1*) [63]. Ace2 is a repressor of the transcription of *CLN3* and its deletion restores the timely expression of this cyclin in the *rts1*∆ mutant. Ace2 is known to be phosphorylated by cyclin-CDK complexes during mitosis in order to block its nuclear import. However, the NDR/LATS family kinase Cbk1 was also shown to phosphorylate Ace2, in this case preventing its nuclear export [103,104]. It is this phosphorylation that PP2A-B56^Rts1^ counteracts, thus facilitating the expression of *CLN3.* Since Cln3-Cdc28 dependent phosphorylation of Whi5 initiates the expression of *CLN1* and *CLN2,* the transcription of these cyclins will be also affected by the dysregulation of Ace2. In addition to this, PP2A-B56^Rts1^ also controls the phosphorylation of Swi4 (which together with Swi6 forms the SBF complex), adding a new layer of regulation of the expression of *CLN1* and *CLN2* during late G1. Reaching a specific level of active Cln3-Cdc28 complex is thought to govern passage through Start and commitment to a new round of division. For this reason, Cln3 was postulated as a dose-dependent modulator of cell size during this phase of the cell cycle. However, the observation that *cln3*∆ cells are still able to regulate their size in response to the nutritional status argues against this model. In contrast to Cln3, PP2A-B56^Rts1^ is required for the modulation of cell size [102]. A possible explanation for the different behaviour of the *cln3* mutant and the *rts1* mutant could stem from the fact that PP2A-B56^Rts1^ controls two different transcriptional pathways (Ace2 and SBF) leading to the expression of early and late G1 cyclins. In the absence of *CLN3*, regulation of the expression of *CLN1* and *CLN2* could still result in size modulation in G1. 

In addition, PP2A-B56^Rts1^ represses the activity of Swe1, which as Cln3 acts as a dose-dependent regulator of cell size during G2. Hence, PP2A-B56^Rts1^ controls the activities of two key elements during G1 and G2. Still, although Cln3 and Swe1 are clearly important to delay cell cycle progression until the cell has grown sufficiently, they are the downstream effectors of molecular pathways that determine cell size. How does PP2A-B56^Rts1^ sense and conveys environmental signals to these regulators? The phenotype of the *rts1*∆ mutant is reminiscent of that of mutants of the PKA pathway or of the AGC kinase Sch9 and the transcription factor Sfp1 [105], which are thought to modulate cell size through the control of the expression of genes involved in ribosome biogenesis. However, it is unclear how the rate of ribosome biogenesis could affect the initiation of the transcription of G1 cyclins, which would determine the time spent in pre-Start G1. Given the similarities between *rts1* and *sch9/sfp1/PKA* mutants, it is possible that PP2A-B56^Rts1^ integrates the rate of ribosome biogenesis and translates it into a quantitative signal that modulates the expression of G1 cyclins. Still, how PP2A-B56^Rts1^ activity is regulated by these pathways remains a question worth investigating in the future.

## 5. Future Perspectives

In recent years, protein phosphatases have raised substantial interest as important elements in the regulation of cell cycle progression, especially as the opposing force to cyclin-dependent kinases. Notably, we have seen that phosphatase activity responds to changes in the nutritional environment resulting in the modulation of cell fate decisions (proliferation/differentiation/quiescence) and the control of cell size. In the future, identification and a better understanding of the specific proteins targeted by these phosphatases will provide further insight onto how they connect the cell cycle machinery to the environmental conditions.
